# Strain-Specific Loci in Bacterial Genomes: Whole-Genome Discovery, Genomic Context, and Application for Multi-Strain qPCR Monitoring

**DOI:** 10.3390/microorganisms14071587

**Published:** 2026-07-21

**Authors:** Emil Elmirovich Valiakhmetov, Mikhail Frolov, Artemiy Yurievich Sukhanov, Aynur Kamilevich Miftakhov, Shamil Zavdatovich Validov

**Affiliations:** 1 Laboratory of Molecular Genetics and Microbiology Methods, Kazan Scientific Center of Russian Academy of Sciences, Orenburgskiy Tract 20A, 420059 Kazan, Russia; e.valiahmetov@knc.ru (E.E.V.); m.frolov@knc.ru (M.F.); a.sukhanov@knc.ru (A.Y.S.); a.miftahov@knc.ru (A.K.M.); 2 Institute of Fundamental Biology and Medicine, Kazan Federal University, 420012 Kazan, Russia

**Keywords:** strain-specific loci, individual strain monitoring, qPCR, TaqMan, GC-context, rhizosphere

## Abstract

Monitoring individual strains in complex microbial communities remains a fundamental challenge in microbial ecology and biotechnology. Here, we present an integrated pipeline for identifying and validating strain-specific loci (SSL) in four biotechnologically relevant plant growth promoting strains from three genera (*Stenotrophomonas*, *Bacillus*, and *Pseudomonas*). The pipeline applies a two-round specificity-filtering strategy combining whole-genome comparison and high-sensitivity BLASTn validation of revealed strain-specific loci (SSL) against the NCBI nucleotide database. SSL count decreased with increasing Average nucleotide identity (ANIb) of the strains used for the analysis, ranging from one locus in *B. halotolerans* (ANIb = 98.91%) to 15 loci in *S. rhizophila* (ANIb = 86.49%). All 25 SSL were universally AT-rich, mainly accessory-genome-associated, with flanking regions enriched in genes of unknown function (34.6%) and mobile genetic elements (19.2%). TaqMan qPCR assays targeting SSL demonstrated high specificity—no target sequences were detected across ten geographically distinct soil samples, nor in a native rhizosphere metagenome—and sensitivity, with limits of detection of 0.01–0.1 pg of genomic DNA. Spike-in experiments in soil yielded method detection limits (MDL) of 850–15,000 CFU/g. All four strains were detected in the wheat rhizosphere seven days after consortium application in a field experiment, validating the pipeline for multi-strain field monitoring.

## 1. Introduction

Monitoring of individual strains is a key challenge in modern microbiology and biotechnology, essential for assessing the persistence of introduced strains in the environment [1,2] or in products derived from biological preparations [3,4] as well as for tracking vaccine strains following immunization [5]. Unlike species-level identification methods based on PCR or isothermal amplification approaches such as LAMP, which target genomic regions conserved across strains of a given species [6,7], detection of an individual strain requires strain-specific loci (SSL)—DNA sequences present exclusively in a single strain, absent in all other strains including the closest phylogenetic relatives, and sufficiently long to serve as qPCR targets.

RAPD, AFLP, rep-PCR, BOX- and ERIC-PCR generate DNA fragment profiles that can be unique to a single strain; however, these fingerprinting methods are applicable to pure cultures only and are unsuitable for detecting individual strains within mixed microbial communities [6].

Microsatellite-containing sequences (simple sequence repeats, SSR; or short tandem repeats, STR) represent another potential source of strain-specific markers: although not unique in themselves, these elements generate unique DNA sequences through their random genomic distribution [8], making them applicable to monitoring individual strains against a background of conspecific strains—as demonstrated, for example, for a biotechnological *Chlorella* strain [3]. In several studies unique sequences have been searched exclusively within open reading frames (ORFs) [4,9]. Andronov et al. (2025) demonstrated the feasibility of identifying SSL in *Rhizobium leguminosarum* strains and successfully applied these sequences as PCR targets [2]. These advances have been enabled by the substantial progress in whole-genome sequencing of microorganisms observed over the past two decades, driven by the declining cost of high-throughput sequencing [10]. The resulting large-scale genomic databases allow comparison of large numbers of genomes, while strain comparison tools such as Tetra Correlation Search (TCS) [11], and Average Nucleotide Identity (ANI)—a metric quantifying the mean identity of orthologous regions between two genomes [12]—enable rapid identification of the closest related genomes.

In the present study, we developed a pipeline for SSL identification and tested it on soil microorganisms representing four different species. Unique loci were identified using KEC [13]. The identified SSL were used to design primers and probes for TaqMan quantitative PCR, one of the most specific detection platforms available [14,15]. The resulting qPCR systems were applied to detect strains in soil and rhizosphere, demonstrating high specificity and sensitivity. In addition, we analyzed the identified SSL sequences to explore their common features, genomic context, and potential functional role in the host genome.

## 2. Materials and Methods

### 2.1. Bacterial Strains and Culture Conditions

The following strains were used in this study: *Stenotrophomonas rhizophila* MGMM118, *Bacillus halotolerans* MGMM119, *Pseudomonas grimontii* MGMM120, and *Pseudomonas viciae* MGMM121, all isolated from the rhizosphere of winter wheat cultivar Universiade. These strains display no mutual antagonism and can be used as a combined biopreparation for plant protection and growth promotion [Patent RU2822893 C1, 15 July 2024]. Strains were stored frozen at −80 °C in phosphate buffer supplemented with 20% glycerol. For routine maintenance, DNA extraction, and preparation of cell suspensions, strains were grown on liquid or solidified King’s B (KB) medium [16]. Since all strains are soil isolates, cultivation was carried out at 30 °C. All four strains were evaluated as a biopreparation in a field trial, providing soil samples that were subsequently used to assess the specificity of the developed qPCR systems.

Whole-genome sequences of the strains were previously obtained and deposited in the NCBI GenBank database under the following accession numbers: *S. rhizophila* MGMM118 (GCF_055520805.1), *B. halotolerans* MGMM119 (GCF_056519215.1), *P. grimontii* MGMM120 (GCF_056682835.1), and *P. viciae* MGMM121 (GCF_056521165.1). Genome characteristics are summarized in Table S1. All four genomes were assembled into a single contig, completeness exceeding 98%, and contamination below 1.2% (Table S1), indicating that the assemblies are suitable for reliable SSL identification.

### 2.2. Identification of Strain-Specific Loci (SSL)

**Figure 1:**
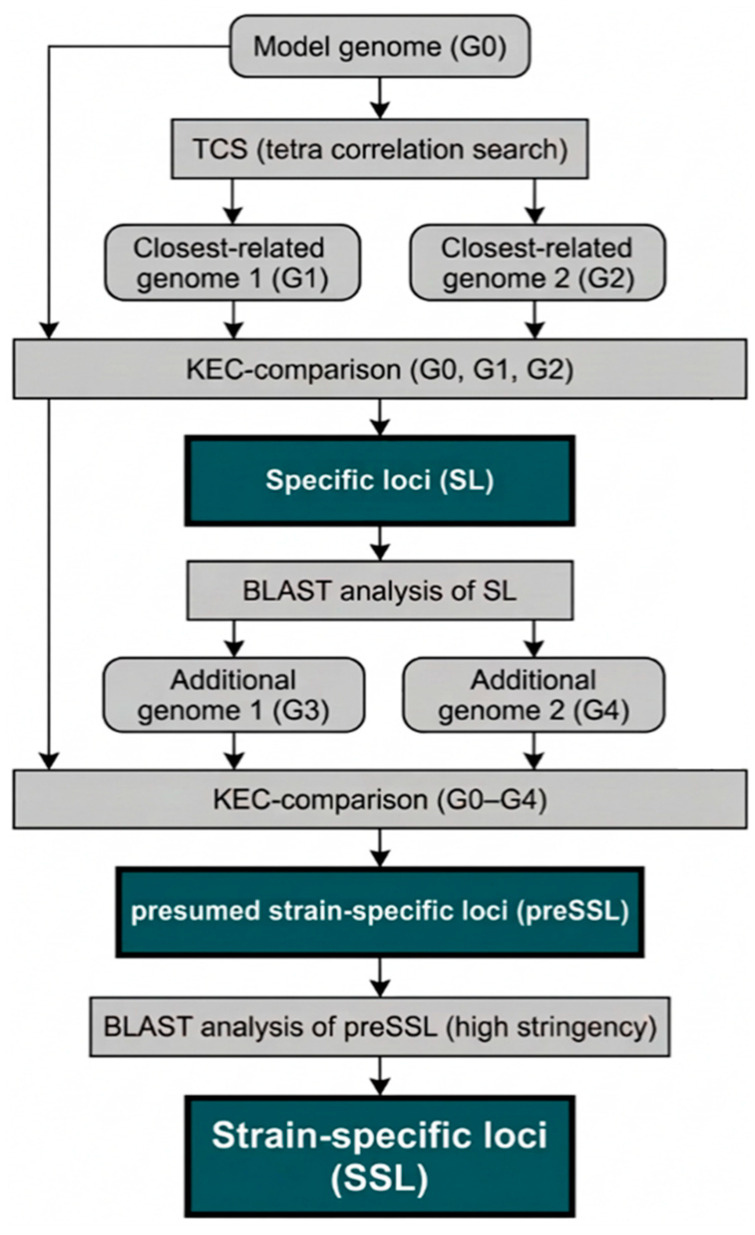
Workflow for the identification of strain-specific loci (SSL). The model (target) genome (G0) is the genome of the target microorganism for which SSL are being identified. G1 and G2 are the two closest-related genomes to G0 as determined by TCS analysis (of sufficient assembly quality). SL (Specific Loci) are loci identified by KEC comparison of the three genomes (G0, G1, G2). G3 and G4 are the genomes most frequently retrieved in BLASTn reports during SL analysis. Presumed Strain-Specific Loci (preSSL) are loci identified by KEC analysis of five genomes (G0 + G1, G2 from TCS + G3, G4 from BLASTn). SSL (Strain-Specific Loci) are loci showing no significant homology to any sequence in the nt database upon high-stringency BLASTn analysis.

The SSL identification workflow is given in Figure 1.

In the first step, the two closest related genomes of each target strain were identified using TCS analysis on the JSpeciesWS [11] platform (version: 5.0.3). To identify fragments of 120–250 bp, the target genome and the two most closely related genomes (G1 and G2) were loaded into KEC (version 1.1), which implements the K-mer Elimination by Cross-reference algorithm [13]. This size range was selected based on the intended downstream application in TaqMan assay design. The choice of two, rather than a larger number of TCS-selected genomes, was determined by empirical tests in which expanding the reference panel reduced the number of candidate loci entering the BLAST stage. The precise reasons for this behavior of KEC with larger panels remain unclear and warrant further investigation; therefore, here we adopt the two-genome configuration as the most efficient and conservative setting identified in our tests. The resulting fragments, designated Specific Loci (SL), were analyzed using BLAST (version 2.13.0) [17] with the following parameters: word size 11, expect threshold 0.05 (BLASTn algorithm against the nt database, https://blast.ncbi.nlm.nih.gov/Blast.cgi, accessed 30 September 2025). For each SL, the genomes containing that sequence were identified, and the two most frequently occurring genomes (G3 and G4) were added to the comparison set alongside G1 and G2.

In the second round, KEC was run with the following parameters:

kec.exe exclude -t [target_dir] -n [nontarget_dir] -o results/test.fasta -k 12 --min 120 --max 250 –r.

-k 12 sets the k-mer size to 12 nucleotides, providing a balance between specificity and sensitivity;

--min 120 and --max 250 restrict the output to sequences between 120 and 250 bp, matching the requirements for qPCR amplicon design;

-r enables the inclusion of reverse-complement k-mers.

The resulting candidate loci (preSSL) were subjected to local BLASTn analysis against the nt database (https://blast.ncbi.nlm.nih.gov/Blast.cgi, accessed 30 September 2025) with the following parameters:

-task blastn-short -word_size 7 -evalue 3 -gapopen 1 -gapextend 1 -reward 1 -penalty -2.

The final SSL set was defined by the absence of significant alignment to any sequence in the nt database, with E-value > 0.05 as the threshold for exclusion, consistent with the default parameter for blastn-short task and the widely accepted significance level in sequence analysis. The reduced word size allows detection of even weak homologies that would be missed with larger k-mer seeds.

### 2.3. Genomic Environment Analysis of SSL

Genomic mapping of SSL onto annotated genomes and GC-content calculations were performed in SnapGene 7 (GSL Biotech LLC, Chicago, IL, USA). We analyzed 2000 bp flanking regions upstream and downstream of each SSL. Functional annotation of genes located in these regions, including genes partially overlapping the window, was carried out using the Pfam database via the InterProScan toolkit (version v5.59-91.0) [18]. Genes were assigned to functional categories based on their annotation (mobile genetic elements, regulation, metabolism, stress response, replication/repair, unknown function, conflict systems). Putative prophage regions in the target genomes were predicted using PHASTEST https://phastest.ca (accessed on 15 October 2025) [19].

### 2.4. BLAST Analysis of Soil Metagenome Samples

To further evaluate the specificity of the SSL, shotgun metagenomic sequencing was performed on rhizosphere DNA from winter wheat plants. DNA was extracted using the Quick-DNA Soil Microbe Microprep Kit (Zymo Research, Irvine, CA, USA) according to the manufacturer’s instructions. DNA concentration was measured with a Qubit 4 fluorometer (Thermo Fisher Scientific, Waltham, MA, USA), and DNA quality was assessed by electrophoresis in a 1% agarose gel. In addition, DNA integrity and fragment size distribution were evaluated using a 2100 Bioanalyzer (Agilent Technologies, Santa Clara, CA, USA) following the manufacturer’s protocol. Libraries were prepared from 100 ng of DNA using the Illumina DNA Prep (Tagmentation) kit, and whole-genome sequencing was carried out on an Illumina NovaSeq X Plus platform with paired-end reads (2 × 151 bp). The overall sequencing quality was Q30 ≥ 93.0% (percentage of bases with Phred score ≥ 30 over the full sequencing run). Demultiplexing was performed with Illumina bcl2fastq v2.20. Adapter trimming was done using Skewer v0.2.2 [20]. Read quality was assessed with FastQC v0.11.5-cegat. Forward and reverse reads were merged into a single dataset and converted from FASTQ to FASTA format using Seqtk https://github.com/lh3/seqtk (accessed on 15 October 2025). Raw metagenomic sequencing reads are deposited in the NCBI Sequence Read Archive under BioProject accession PRJNA1473945 (SRA: SRS29397332). BLASTn database was built from the resulting file, and SSL sequences were searched against this database using highly sensitive parameters described above in Section 2.2. The same high-sensitivity BLASTn settings were used as in the final in silico SSL filtering step, with an E-value significance threshold of 0.05 for interpreting hits.

### 2.5. DNA Manipulation and Polymerase Chain Reaction (PCR)

#### 2.5.1. Sampling, Extraction, and Quantification of DNA

Genomic DNA from bacterial cultures was extracted using the HiPure Bacterial DNA Kit (Magen Biotechnology, Guangzhou, China) according to the manufacturer’s instructions. Total DNA from soil samples was extracted using the HiPure Soil DNA Kit (Magen Biotechnology, Guangzhou, China) from 0.4 g of soil per sample, following the manufacturer’s protocol. DNA concentrations were determined fluorometrically on a Qubit 4 fluorometer (Thermo Fisher Scientific, Waltham, MA, USA) using the QuDye dsDNA BR Assay Kit (Lumiprobe, Moscow, Russia).

#### 2.5.2. Primer Design, PCR, and Electrophoresis

Primers and TaqMan probes for the detection of *S. rhizophila* MGMM118, *B. halotolerans* MGMM119, *P. grimontii* MGMM120, and *P. viciae* MGMM121 were designed in SnapGene 7.0 (GSL Biotech LLC, Chicago, IL, USA) (Table 1) based on the SSL identified for each strain (Table S2). SSL were selected according to the following criteria: maximal uniqueness (based on E-value) in BLASTn analysis, preferential location outside annotated coding sequences (intergenic or spanning CDS boundaries rather than fully embedded within a CDS) and suitability for designing highly specific oligonucleotides with high melting temperatures (Tm ≥ 57 °C) that do not form dimers, secondary structures, or cross-hybridizing interactions.

The PCR mixture (20 µL total volume) contained 5× HS qPCR-mix (Evrogen CJSC, Moscow, Russia), 10 ng of total DNA, and 0.5 µM of each primer. Amplifications were performed on a QuantStudio 5 real-time PCR system (Thermo Fisher Scientific, Waltham, MA, USA). PCR products were analyzed by electrophoresis in 1.5% agarose gels prepared in 1× TAE buffer (Evrogen CJSC, Moscow, Russia). DNA fragments were excised from the gel and purified using the CleanUp kit (Evrogen CJSC, Moscow, Russia). Nucleotide sequences of the amplified fragments were determined by Sanger sequencing at Evrogen company (Moscow, Russia).

#### 2.5.3. qPCR for Sensitivity and Specificity Testing

TaqMan qPCR reactions were prepared using 5× qPCRmix-HS LowROX (Evrogen CJSC, Moscow, Russia), with 0.5 µM probe, 0.5 µM each forward and reverse primer, 2 µL of DNA template at the desired concentration, and nuclease-free water to a final volume of 20 µL. For assays with DNA from pure cultures, 20 ng of DNA was added per reaction. Amplification was carried out on a QuantStudio 5 real-time PCR system (Thermo Fisher Scientific, Waltham, MA, USA) under the following conditions: initial denaturation at 95 °C for 5 min, followed by 40 cycles of 95 °C for 20 s and 59 °C for 60 s with fluorescence acquisition at the annealing/extension step. All experiments included no-template controls.

To construct standard curves, ten-fold serial dilutions of purified genomic DNA (100–0.01 pg per reaction) were prepared for each strain and amplified in triplicate. Standard curves were generated by plotting Cq values against log_10_(DNA amount, pg). The amplification efficiency (E) was calculated from the slope of the standard curves. All qPCR data were analyzed using QuantStudio Design & Analysis Software 2.5.1 (Thermo Fisher Scientific, Waltham, MA, USA) and MS Excel (Microsoft, Redmond, WA, USA).

The limit of detection (LOD) for purified genomic DNA was defined as the lowest DNA amount per reaction that yielded a positive amplification signal in all technical replicates.

DNA quality and the absence of PCR inhibitors for specificity tests were assessed using primers U789 and U1053 (0.5 µM each), which target the 16S rRNA gene. Reactions were carried out with the 5 × qPCRmix-HS SYBR + LowROX kit (Evrogen CJSC, Moscow, Russia) under the following conditions: 95 °C for 5 min, followed by 40 cycles of 95 °C for 20 s and 52 °C for 60 s with fluorescence acquisition. All experiments included no-template controls.

### 2.6. Assessment of SSL Transcriptional Activity

To evaluate SSL transcription levels, bacterial cultures were grown in liquid KB medium to stationary phase (16–18 h, OD_600_ ≈ 1.5–2.0). Total RNA was extracted from 2 mL of culture using the RNA-Solo kit (Evrogen CJSC, Moscow, Russia) according to the manufacturer’s protocol. Residual genomic DNA was removed by treatment with DNase I (Evrogen CJSC, Moscow, Russia), followed by RNA clean-up on CleanRNA Standard spin columns (Evrogen CJSC, Moscow, Russia). RNA concentration and purity were determined spectrophotometrically with a NanoDrop One instrument (Thermo Fisher Scientific, Waltham, MA, USA).

cDNA was synthesized using MMLV reverse transcriptase (Evrogen CJSC, Moscow, Russia) in 20 µL reactions containing 500 ng of total RNA, 5 µM primer mix, 0.5 mM of each dNTP, 1× reverse transcriptase buffer, and 100 U MMLV. For each strain, primers specific to the corresponding SSL and to the reference gene *rsfS* (Table 1) were used. Reverse transcription was performed at 42 °C for 60 min, followed by enzyme inactivation at 70 °C for 10 min. To rule out contamination with genomic DNA RNA preparations without reverse transcription were also used as a PCR templates (no-RT controls).

Quantitative PCR was performed on a QuantStudio 5 system, each 20 µL reaction contained 2 µL of cDNA (five-fold diluted), 0.4 µM of each primer, and 4 µL of 5× qPCRmix-HS SYBR + LowROX (Evrogen CJSC, Moscow, Russia). The cycling conditions were as follows: initial denaturation at 95 °C for 3 min, followed by 40 cycles of 95 °C for 15 s and 60 °C for 1 min, with fluorescence recorded during the annealing/extension step. All measurements were performed in three biological and three technical replicates.

SSL expression levels were evaluated relative to the reference gene *rsfS*, which is a single-copy gene in all studied strains and exhibits low, constitutive expression in stationary phase. For each SSL-*rsfS* pair, ΔCq was calculated as Cq(SSL) − Cq(*rsfS*). Relative expression was calculated as a percentage of *rsfS* expression using the formula 2^(−ΔCq)^ × 100%. Because relative expression 2^(−ΔCq)^ follows a log-normal distribution, its uncertainty is expressed as the asymmetric interval 2^−(ΔCq±SD(ΔCq))^, rather than as a symmetric standard deviation.

### 2.7. Method Detection Limit

To assess the detection threshold and to obtain strain-specific calibration coefficients for soil samples, a spike-in experiment was performed. For each strain, 40 µL of bacterial suspension with optical densities of OD_600_ = 0.01, 0.001, and 0.0001 were added to 400 mg aliquots of non-sterile field soil. Three independent replicate tubes were prepared for each dilution. Total DNA was immediately extracted using the HiPure Soil DNA Kit (Magen Biotechnology, Guangzhou, China) according to the manufacturer’s protocol. DNA was eluted in 50 µL of elution buffer, then brought to a final volume of 200 µL with nuclease-free water. Two microlitres of the diluted DNA were used as template in a 20 µL qPCR reaction.

Simultaneously, the exact Colony Forming Units (CFU) corresponding to optical density was determined by plating serial dilutions of the same bacterial suspensions onto KB agar in triplicate.

The number of genome equivalents detected by qPCR in each soil sample was determined from the measured Cq values using the strain-specific calibration curves obtained with purified genomic DNA (see Section 2.5.3). For each strain and each dilution, a calibration coefficient (genome copies per CFU) was calculated as:

Genome copies per CFU = (qPCR-measured genome equivalents per g soil)/(inoculated CFU per g soil).

This coefficient reflects the combined efficiency of DNA extraction and qPCR amplification for a given strain in the soil matrix. A mean calibration coefficient (±SD) was obtained for each strain by averaging the values from the three dilutions. The method detection limit (MDL) was defined as the lowest inoculated CFU/g soil at which the target DNA was detected in all biological replicates.

### 2.8. Field Experiment on Winter Wheat

Winter wheat plants at the tillering stage were treated with a consortium-based biopreparation comprising four target strains (MGMM118, MGMM119, MGMM120, MGMM121). Bacterial suspensions were prepared in sterile phosphate-buffered saline (PBS, pH 7.4) at OD_600_ = 0.1 for each strain. The application rate was 100 mL of suspension per square meter. Rhizosphere soil for control samples was collected before biopreparation application. Seven days after treatment, rhizosphere soil samples were collected from three plants. DNA extraction and TaqMan qPCR for soil samples and pure cultures were performed as described in Section 2.5.3 (0.4 g).

For each sample, the amount of target DNA in reaction was determined from the measured Cq value using the standard curve (Section 2.5.3). The total amount of target DNA extracted from the soil sample was then calculated by multiplying this value by the ratio of the total volume of the eluate (300 µL) to the template volume (2 µL):

CFU/g soil = (DNA_(pg)_ × V_total_/V_template_)/(m_genome_ × m_soil_ × k)

DNA_(pg)_—amount of target DNA (pg) determined from the standard curve;

V_total_—total volume of the diluted soil DNA eluate (in our case 300 µL);

V_template_—volume of template added to each qPCR reaction (2 µL);

m_genome_—mass of a single genome copy (pg);

m_soil_—mass of the soil aliquot used for DNA extraction (0.4 g);

k—strain-specific calibration coefficient (genome copies per CFU) determined in the spike-in experiment.

## 3. Results

### 3.1. Identification of Strain-Specific Regions in Whole-Genome Sequences

Strain-specific loci (SSL) were revealed for four bacterial strains belonging to the genera *Stenotrophomonas*, *Bacillus*, and *Pseudomonas*. The smallest genome size (4.07 Mbp) was observed for *Bacillus halotolerans* MGMM119, while *Stenotrophomonas rhizophila* MGMM118 had a comparable genome size of 4.17 Mbp. The two strains of fluorescent pseudomonads, *Pseudomonas grimontii* MGMM120 and *P. viciae* MGMM121, had genome sizes of 6.37 Mbp and 6.46 Mbp, respectively. In *S. rhizophila* MGMM118 and *B. halotolerans* MGMM119, 3666 and 3931 coding sequences (CDS) were annotated, whereas the two pseudomonas strains carried 5701 and 5571 CDS, respectively (Table S1). The genome of *B. halotolerans* MGMM119 was characterized by a relatively low GC content (43.8%), while *P. grimontii* MGMM120 and *P. viciae* MGMM121 had higher GC contents (60.7%). The genome of *S. rhizophila* MGMM118 was the most GC-rich (67.1%). Thus, the set of strains used for SSL discovery represented phylogenetically diverse bacteria whose genomes differed in size, CDS content, and GC composition.

To identify SSL, we applied the workflow given in Figure 1 Among the 100 closest genomes returned by TCS for each target, the group associated with *B. halotolerans* MGMM119 showed the highest mean Z-score (0.9969), indicating a high density of closely related genomes in open databases. In contrast, the *S. rhizophila* MGMM118 group had the lowest mean Z-score (0.9711), reflecting a lower representation of closely related genomes in the databases. The 100-genome sets for *P. grimontii* MGMM120 and *P. viciae* MGMM121 were characterized by intermediate mean Z-scores of 0.9945 and 0.9849, respectively. Based on these results, we selected two genomes per target strain that exhibited the highest Z-scores relative to the corresponding target, i.e., those most similar in tetranucleotide composition.

The number of SL revealed in the genomes of target strains varied from 96 in *B. halotolerans* to 111 in *S. rhizophila* strains (Table 2). BLASTn analysis of the SL showed that most loci were also present in genomes of strains closely related to the corresponding target strains.

K-mer-based analysis using KEC was performed on groups uniformly composed of the target strain, two TCS-derived strains, and two BLAST-derived strains (Table S3). Among these, the *S. rhizophila* MGMM118 group, which exhibited the lowest similarity metrics (ANIb 86.49% and aligned genome fraction 72.90%), yielded the highest number of candidate loci, with 20 preSSL and 15 SSL retained after final filtering. In contrast, the *B. halotolerans* MGMM119 group, characterized by high genomic similarity (ANIb/aligned fraction = 98.91/94.58), produced only a single preSSL, which was retained as an SSL after filtering (Table 2). The *P. grimontii* MGMM120 and *P. viciae* MGMM121 groups showed intermediate values for both ANIb and aligned genome fraction, with similarity metrics comparable to those of their closest relatives (Table 2). Consistent with this, KEC identified similar numbers of preSSL and SSL in these two *Pseudomonas* groups.

### 3.2. Analysis of SSL Genomic Environment

To identify common features of SSL across the four strains, we analyzed the relationship between SSL and the functional genome organization. For each SSL and its 2 kb flanking regions on both sides genomic context was examined in terms of annotated genes, predicted prophage regions, and local GC content.

Of the 25 SSL analyzed, 12 were located entirely within coding regions (CDS), 4 entirely within non-coding regions, and 9 spanned CDS boundaries, partially covering both coding and non-coding sequences (Table S2). We also calculated the fraction of coding sequence for each SSL together with its flanking regions and obtained a mean value of 79.2 ± 11.2%. Thus, neither the distribution of SSL between coding and non-coding regions nor the average proportion of coding sequence in SSL plus flanks differed markedly from the corresponding genome-wide values. Given that coding sequences typically occupy more than 80% of bacterial genomes overall, we conclude that SSL do not show a strict association with either coding or non-coding regions.

Although all four genomes contained confidently predicted prophage regions, only a single SSL (no. 21) was located within a prophage-associated sequence (Table S2). This indicates that the identified SSL are generally not associated with prophage regions.

For each flanking region, we analyzed the local functional genomic context by extracting all annotated ORFs overlapping the ±2 kb window (genes were included even if they only partially overlapped the region). Analysis of all SSL-flanking regions showed that the detected genes could be assigned to five functional categories (Table 3): 34.6% of the genes were of unknown function, 23.1% of the genes were annotated as involved in cellular metabolism, 19.2% of the genes were associated with mobile genetic elements, 14.1% of revealed genes were annotated as belonging to regulation and signaling. The least represented category comprised genes associated with DNA replication, repair, and modification (9%).

From the entire set of genes with predicted functions, those annotated as RHS repeat-associated protein, C4-antisense RNA, and DNA adenine methylase were found in regions flanking different independent SSL (Table S4). Notably, all of these genes are associated with bacterial defense and antagonism (“conflict systems”), which were frequent in the genomic context of SSL. For example, components of restriction–modification systems (DNA adenine methylase, EcoRII-like endonuclease), two RHS-associated proteins, three C4-antisense RNA genes, as well as HNH/NHH endonucleases, anti-phage toxin modules (HEPN/NYN and caspase-like proteins), and a bacteriocin-like gene (colicin E3/pyocin S6 family) were independently detected near different SSL.

Nucleotide composition analysis showed that all revealed SSL were AT-rich regions and consistently had lower GC content than the corresponding host genomes (Table S2). The GC content of the flanking regions was also lower than the genome average, although the difference was less pronounced. The only exception was the flanking region of *B. halotolerans* MGMM119, which was even more AT-rich than its SSL (Table 4).

### 3.3. Selection of Target Regions and Primer Design for qPCR

For use as qPCR markers, one locus was finally selected for each target strain (sequences are listed in Table S2). The selection criteria were chosen to maximize the specificity and performance of the qPCR assays. First, preference was given to loci located fully or partially in non-coding regions and showing the lowest best-hit E-values in BLASTn. Second, an important practical constraint was the feasibility of designing a multiplex “primer–probe–primer” set in the vicinity of the locus, with high melting temperatures and low dimerization propensity of the oligonucleotides. During primer design, shifts of up to 100 bp from the original SSL sequence position were made when necessary. The resulting amplicon regions (Table S5) were subsequently validated using BLASTn. To further increase expected qPCR specificity, primers were designed to have an annealing temperature of at least 55 °C (Table 1). Capillary Sanger sequencing of the amplicons confirmed complete identity with the in silico SSL-derived sequences for each strain.

### 3.4. Transcriptional Activity of Selected SSL

**Figure 2:**
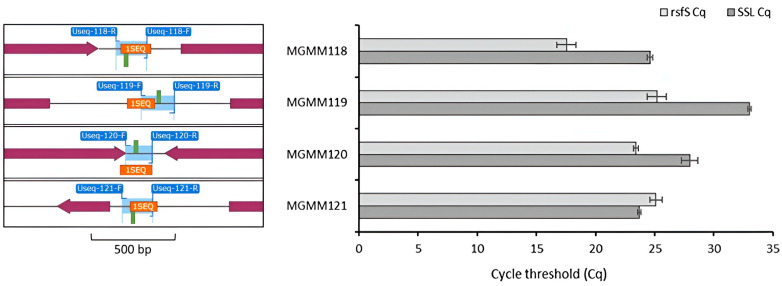
Transcriptional activity of selected SSL. **Left**: Schematic representation of the genomic regions harboring selected SSL. Maroon blocks indicate annotated genes, orange blocks mark the SSL, green blocks indicate TaqMan probes binding sites, and blue arrows/blocks denote the binding sites of the designed primers. The amplicons, which span the region between primers and encompass the probe binding site, are shown as blue blocks (scale bar: 500 bp). **Right**: Quantification cycle (Cq) values for SSL and the reference gene *rsfS* of each strain, shown as mean ± SD.

To estimate transcription levels of the selected SSL, quantitative RT-qPCR was performed using the same primers specific to each SSL (Table 1) together with universal primers for the reference gene *rsfS* (Figure S1). The results of the measurements are presented in Table 5 and visualized in Figure 2.

SSL expression levels varied between strains and depended on the position of each SSL relative to annotated genes (Figure 2). Although all four SSL are annotated as non-coding regions, they occupy different genomic positions with respect to neighboring genes and display distinct transcriptional levels. The highest relative expression was observed for the SSL in strain MGMM121 (266% of *rsfS*); this locus is located within 100 bp of the start codon of the adjacent gene and thus falls into its putative 5′ untranslated region. In strain MGMM118, SSL expression was substantially lower (0.75% of *rsfS*); here, the locus lies in an intergenic region directly adjacent to annotated coding sequences. In strain MGMM120, the SSL showed a relative expression level of 4.2%; this locus partially overlaps (by 5 bp) the stop codon of the neighboring gene while being otherwise intergenic. Finally, the SSL of strain MGMM119 exhibited the lowest relative expression among all strains (0.45% of *rsfS*); this locus is located far from the nearest predicted coding regions (>500 bp).

### 3.5. Sensitivity Assessment

To determine the analytical sensitivity of the qPCR systems with purified DNA, ten-fold serial dilutions of genomic DNA from each of the four strains were prepared in the range from 100 to 0.01 pg per reaction. Within the range of 100 to 0.1 pg, all assays showed a strong linear relationship between DNA concentration and Cq values (R^2^ ≥ 0.9986; Efficiency ≥ 88.5%, Table S6 and Figure S2). The limit of detection (LOD) defined as the lowest DNA amount that consistently yielded a positive amplification signal for purified genomic DNA was set at 0.01 pg per reaction for strains MGMM118, MGMM119 and MGMM121, and at 0.1 pg for MGMM120 (Table 6). At these LOD levels the estimated numbers of genome equivalents ranged from approximately 1 to 14, depending on the strain and its genome size.

Because the intended application of the assays is the monitoring of strains in soil, we further assessed the detection sensitivity in soil by spike-in experiments. Serial dilutions of cell suspensions of each target strain were added to non-sterile soil samples, followed by total DNA extraction and qPCR. For each strain a conversion factor—the number of genome copies detected per inoculated CFU—was determined from the spike-in data and used to relate qPCR-measured genome equivalents to CFU. The method detection limit (MDL) was defined as the lowest CFU/g soil at which a positive amplification signal was obtained in all three replicates. The MDL values and the corresponding genome-copy-per-CFU ratios are shown in Table 7. The MDL ranged from 6.5 × 10^3^ CFU/g for MGMM119 to 1.5 × 10^4^ CFU/g for MGMM120. Genome copies per CFU varied between 0.48 and 2.12.

### 3.6. Specificity Testing

DNA extracted from soil contains a highly heterogeneous mixture of nucleotide sequences that can be used to rigorously assess the specificity of primers for PCR-based monitoring. To test assay specificity, total DNA was extracted from soil samples collected at ten different locations, including sites where the four strains were isolated and arbitrarily chosen soil samples stored frozen in the laboratory (Table S8). No amplification products were detected in any of these soil DNA samples. The absence of detectable amplification when using DNA isolated from soils indicates high primer specificity even in the presence of a large excess of heterologous DNA. To exclude false-negative results in this test, qPCR with universal primers targeting the V3–V4 region of the 16S rRNA gene was performed using SYBR Green. This control ruled out poor DNA quality or PCR inhibition. The resulting Cq values ranged between cycles 15 and 18, consistent with a high bacterial DNA content in the samples and indicating no significant inhibition of the PCR.

### 3.7. Search for SSL in Metagenomics Data

Analysis of shotgun metagenomes obtained from rhizosphere DNA of winter wheat plants did not reveal any significant matches to any of the 25 SSL. Thus, SSL uniqueness was confirmed not only relative to public databases and bulk soil DNA, but also directly within the rhizosphere metagenome of the experimental plots later used for field monitoring experiment.

### 3.8. Detection of Strains in the Field Experiment

**Figure 3:**
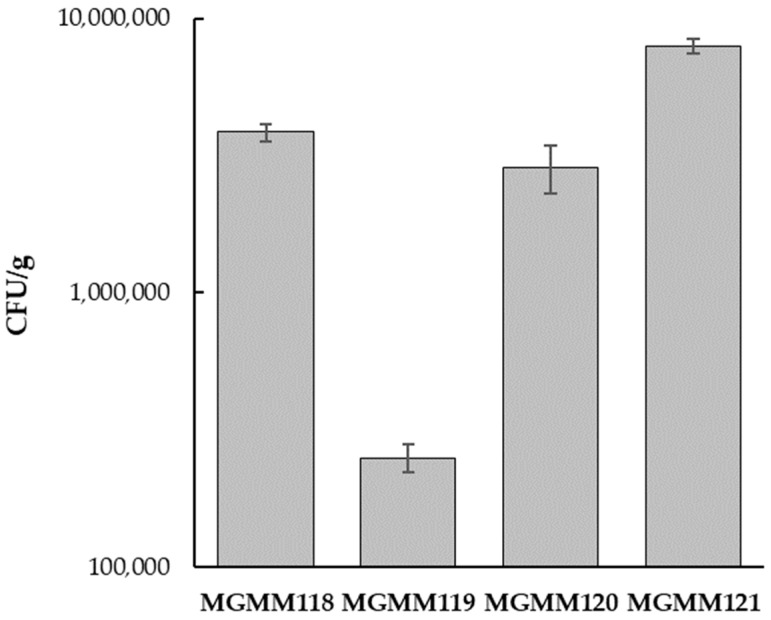
Detection of four target strains in winter wheat rhizosphere seven days after application of the biopreparation. Bars represent the calculated CFU per gram of soil (Table 8).

The SSL-based detection and monitoring system was further validated under field conditions. In rhizosphere samples collected seven days after foliar application of the four-strain consortium, all four strains were detected, whereas no amplification of SSL from any of the model strains was observed in the control soil sampled prior to treatment (Figure 3; quantitative data are provided in Table 8).

All four strains were detected in the rhizosphere (Figure 3, Table 8). The estimated colonization levels ranged from 2.5 × 10^5^ to 8.0 × 10^6^ CFU/g, exceeding the conservatively defined MDL (Table 7) by one to three orders of magnitude. Together, these data demonstrate that the SSL-based qPCR systems are suitable for monitoring of a multi-strain bacterial consortium under realistic field conditions.

## 4. Discussion

Monitoring individual strains is an important task in biotechnology and microbiology, as reflected by the growing number of publications and software packages dedicated to identifying highly specific DNA targets that can be used to build assays for detecting single strains within complex microbial communities [1,2,3]. The most straightforward way to search for unique fragments would be to compare all available genome sequences. However, exhaustive information on all existing sequences is only a theoretical ideal. In practice, the discovery of truly strain-specific loci requires algorithms that operate on a limited set of genomes. This limitation reflects a broader challenge: for many environmentally important bacteria, the poor representation in public databases constrains our ability to identify strain-specific loci. While our pipeline has been validated on strains with contrasting genome sizes and GC content, its applicability to other taxonomic groups, especially those with high intraspecific variability or equally limited database representation, remains to be tested.

Highly automated tools such as NAUniseq [26] and Fur [27] work at the k-mer level; however, NAUniseq is restricted to in silico evaluation of specificity, while Fur generates unique fragments for a pre-defined set of closely related genomes and does not guarantee strain-level specificity in complex microbial communities. Despite being constrained to coding sequences, PUPpy and UniOrtho, as well as the approach described by Hernández et al., have shown high practical utility for designing TaqMan qPCR systems capable of strain detection in soil and plant samples [3,4,8].

Conceptually, our work is also related to approaches based on assembling unique contigs from unmapped WGS reads [28], where the authors similarly performed a brief analysis of the genetic environment of the identified loci. However, in that study, specificity was evaluated only in silico, without in vitro testing. In Andronov et al. (2025), strain-specific fragments were identified for a single species and not tested against rhizosphere community DNA, although the work is accompanied by a thoughtful discussion of the origin of unique fragments and their genetic context [2].

Our pipeline differs from these approaches in several key aspects. First, we abandoned an ORF-oriented strategy focused on polymorphism in pre-defined loci (e.g., those associated with mobile genetic elements) or conserved genes, and instead treated the genome as a continuous nucleotide sequence. This enables the discovery of unique loci in intergenic regions. Second, we implemented a two-round filtering scheme: after an initial selection of loci by KEC on the target genome (G0 in Figure 1) with two TCS-selected closely related genomes (G1, G2), we explicitly searched for strains (G3, G4) that partially carried the same sequences and added them to the second comparison round. This allowed us to “target” the comparison panel to the nearest neighbors that differ by a minimal number of genomic rearrangements. Third, the final BLAST-based filtering was performed with high-sensitivity parameters against the nt database, greatly reducing the chance of missing weak but biologically meaningful homology. It is worth noting that all SSL discovery approaches, including ours, critically depend on the quality of the target genome assemblies. As sequencing technologies continue to improve and high-quality reference genomes become more widely available, the reliability of SSL identification is expected to increase accordingly.

Applying this pipeline to four strains from *Stenotrophomonas*, *Bacillus*, *Pseudomonas* genera revealed a marked difference in the number of final SSL depending on the genomic relatedness between the target strain and the non-target genomes in the comparison panel. For *B. halotolerans* MGMM119 (ANIb = 98.91%, Aligned = 94.58%), only a single SSL was identified; for *S. rhizophila* MGMM118 (ANIb = 86.49%, Aligned = 72.90%), 15 SSL were found. The *P. grimontii* and *P. viciae* groups occupied an intermediate position in terms of both ANIb and SSL count. While based on only four strain groups, this qualitative observation is consistent with the view that strain-specific loci belong to the most rapidly diverging part of the bacterial genome: within the pangenome concept, unique sequences constitute that portion of the accessory and variable regions that predictably declines as genome similarity to the closest characterized relatives increases [29]. Importantly, this pattern is unlikely to be an artifact of unequal taxon representation in public databases. Indeed, the genus *Pseudomonas* has the largest number of sequenced genomes (>23,000 complete assemblies), yet the number of SSL in our pseudomonad strains is not minimal. Conversely, the genus *Bacillus* takes an intermediate position by genome count (>13,000 assemblies) but showed the lowest SSL count—only one locus was revealed in *B. halotolerans*. Even though ANIb values may partly reflect the density of genome sampling in a given taxonomic group, the results suggest that the primary factor determining the number of identifiable unique loci is not only database size *per se*, but also the biological divergence between the target strain and its closest sequenced relatives.

Beyond the differences in SSL counts, we analyzed qualitative properties of SSL and their genomic environments. All 25 SSL were AT-rich relative to their host genomes, and this trend was evident even in *B. halotolerans* MGMM119, whose genome is overall GC-poor. AT-rich regions are known to have reduced thermodynamic stability, to undergo local unwinding more frequently, and to serve as preferred targets for the integration of mobile genetic elements and non-homologous recombination events [30,31]. Furthermore, spontaneous mutational processes in bacteria are universally biased toward A/T [32]. Thus, the observation that all SSL, identified by comparison of closely related genomes, are localized in potentially unstable regions, prone to rapid accumulation of changes, suggests that our pipeline is effective for identifying rapidly evolving and therefore unique DNA fragments.

According to the predicted functions of the genes discovered in the regions flanking all 25 SSL 34.6% were the genes of unknown function, 19.2% of the genes were associated with mobile genetic elements although putative association with prophages was predicted only 1 SSL in MGMM120. Genes annotated as DNA adenine methylase, C4-antisense RNA, RHS repeat-associated protein, and HNH-type nuclease occurred independently in the flanking regions of different SSL in different strains. These components are often classified as parts of bacterial “conflict systems”, which have been shown to be consistently associated with mobile elements and recombination hotspots [33,34]. Such genetic surrounding of the SSL may be classified as regions of the accessory genome, where selective constraints are relaxed and high genetic variability is tolerated. Taken together, these features support the view that SSL uniqueness is not an artifact of database incompleteness, but is determined by the intrinsic properties of the most variable regions of bacterial genomes.

Turning to the functional properties of SSL, it is noteworthy that, despite the deliberate selection of loci outside extended coding regions for use as qPCR targets, quantitative RT-qPCR detected transcription of all four selected SSL in the stationary phase. Relative transcript levels normalized to the reference gene *rsfS* spanned a wide range, from 0.45% in MGMM119 to 266% in MGMM121, and the differences between the highest and lowest values were robust to technical variation (Table 5, Figure 2). This variability was associated with the distance from SSL to the nearest annotated gene. The highest mRNA level was observed for the SSL of MGMM121, located within 100 bp of the start codon of the adjacent gene and presumably overlapping its 5′ regulatory region. Intermediate values (0.75–4.2%) were recorded for the SSL in MGMM118 and MGMM120, which are partially adjacent to coding sequences. The lowest transcription (0.45%) was detected for the SSL of MGMM119, located more than 500 bp away from the nearest predicted start codons.

It is plausible that the generally low transcription detected for SSL reflects not only their location distal to active coding sequences, but also the involvement of xenogeneic silencing—a dedicated mechanism by which nucleoid-associated proteins such as H-NS bind and transcriptionally repress AT-rich DNA regions, many of which are of horizontally transferred origin [35,36]. The proposed biological function of this mechanism is to limit the potentially harmful expression of newly acquired genetic material until it becomes integrated into existing regulatory networks. In this framework, the low SSL expression levels are an expected outcome of their AT-rich, noncoding nature rather than an incidental feature. MGMM121 seems like a notable exception: both its absolute GC content and its deviation from the genomic average are less extreme than for the other SSL, and its overlap with the 5′ regulatory region of an active gene locally counteracts H-NS-mediated silencing.

Beyond these fundamental aspects, the practical utility of the developed systems critically depends on their specificity, which we verified at several levels. First, the SSL identified in this study showed no significant homology to any sequence in the nt database when screened with high-sensitivity BLASTn parameters. Second, none of the four SSL fragments mapped significantly to metagenomic sequences obtained from rhizosphere soils of experimental fields where various biopreparations and crops have been tested over multiple years. Third, TaqMan qPCR assays targeting each SSL produced no signal when applied to total DNA extracted from ten geographically distinct soil samples collected both at the strain isolation site and at distances of 0.5–1 km, as well as from other regions of Russia and abroad. This three-level, orthogonal validation sets our work apart from most previous studies, in which specificity was assessed solely in silico [26,28] and supports the conclusion that the selected SSL are genuinely unique with respect to the natural microbial diversity accessible for analysis at present.

Alongside specificity, assay sensitivity is a critical parameter. The developed TaqMan qPCR systems showed high analytical sensitivity: on purified genomic DNA the limit of detection (LOD) was 0.01 pg per reaction for strains MGMM118, MGMM119, and MGMM121, and 0.1 pg for MGMM120, corresponding to approximately 1–14 genome equivalents per reaction depending on genome size. In soil matrix experiments, the method detection limit (MDL) predictably increased by 2–3 orders of magnitude due to incomplete DNA extraction, ranging from 6.5 × 10^3^ CFU/g (MGMM119) to 1.5 × 10^4^ CFU/g (MGMM120) (Table 7). These values are at or below the typical detection thresholds for shotgun metagenomics (~10^4^–10^6^ CFU/g for a target taxon) [37], while qPCR is substantially faster, less expensive, and provides strain-level resolution. The same sensitivity threshold likely explains the absence of qPCR signals in soil samples collected three years after the original isolation of the strains: given that the strains were obtained using a root-colonization enrichment system, it is highly probable that *S. rhizophila* MGMM118, *B. halotolerans* MGMM119, *P. grimontii* MGMM120, and *P. viciae* MGMM121 do not persist at a detectable number in bulk soil outside the rhizosphere.

Strain-specific calibration coefficients (genome copies per CFU) ranged from 0.48 to 2.12. The value exceeding unity observed for *P. grimontii* MGMM120 (k = 2.12) is consistent with oligoploidy, which has been documented in *Pseudomonas* where up to 20 genome copies per cell have been reported [38]. Notably, despite the lower DNA extraction efficiency typically associated with Gram-positive bacteria due to their rigid cell wall, the coefficient for *B. halotolerans* MGMM119 (k = 0.48) exceeded our expectation for a Gram-positive organism. This may partly reflect a degree of oligoploidy that partially compensates for incomplete cell lysis, in line with reports of oligoploidy in *Bacilli*, where chromosome copy numbers of 6–12 per cell have been observed during exponential growth [39]. Importantly, since bacterial ploidy rarely exceeds tens of genome copies, this effect introduces at most a one-order-of-magnitude offset in CFU estimates and does not compromise the utility of the assay for field monitoring, where detected population densities exceeded the MDL by one to three orders of magnitude.

Detection of all four strains in the wheat rhizosphere seven days after a single foliar application of the consortium confirmed that the SSL-based TaqMan qPCR systems perform robustly under realistic field conditions. Estimated colonization levels ranged from 2.5 × 10^5^ to 8.0 × 10^6^ CFU/g. Although the absolute CFU/g values should be viewed as estimates, the data clearly demonstrate active rhizosphere colonization well above the detection threshold. Together, these results validate SSL-based qPCR as a sensitive and quantitative tool for simultaneous monitoring of multi-strain microbial consortia in agricultural settings, with strain-level specificity.

The SSL status of a locus is dependent on the current content of public nucleotide sequences repositories, meaning that any locus considered to be present in a single strain today may lose its uniqueness once it is discovered in a newly deposited genome of another strain. We view this fallibilistic uniqueness of SSL as an intrinsic property of the methodology rather than a flaw, and one that necessitates periodic re-evaluation. Our pipeline explicitly accommodates such re-evaluation by allowing repeated BLAST validation of SSL whenever the nucleotide databases are substantially updated. Beyond database dynamics, the long-term stability of SSL is also constrained by evolutionary processes: bacterial base-substitution rates are typically on the order of 10^−10^ per site per generation [40,41], implying that SSL regions of 120–250 bp are expected to accumulate substantially fewer than one substitution over tens to hundreds of generations in the absence of strong selection. In contrast, largescale events such as recombination and horizontal gene transfer are sporadic and highly context-dependent, so reliable locus-specific predictions remain challenging. Therefore, for long-term or multi-year field monitoring, it is important to keep in mind that evolutionary changes in the target genome may affect the interpretation of results. From a practical standpoint, however, the SSL identified here have already demonstrated sufficient specificity to enable confident detection of target strains against the background of complex natural microbial communities, whose taxonomic composition is independent of ongoing changes in sequence databases.

## 5. Conclusions

In this study, we proposed an experimentally validated pipeline for identifying strain-specific loci based on whole-genome KEC search, two-round filtering with inclusion of BLAST-selected relatives, and high-sensitivity BLASTn validation. Using four biotechnologically relevant strains from three genera as a test case, we show that the number of final SSL is primarily determined by intraspecific biological divergence, which we can currently infer only from available genome sequences of related strains. All SSL identified in this work are predominantly AT-rich segments of the accessory genome characterized by low transcriptional activity, which may indicate accelerated evolution towards uniqueness under relaxed selective pressure.

TaqMan qPCR systems designed on the basis of these SSL passed three levels of specificity validation and enabled reliable detection of all four strains in wheat rhizosphere under field conditions. In terms of specificity and sensitivity, the proposed approach is comparable to metagenomic methods, yet it is substantially faster and more cost-effective, making it a promising tool for routine strain monitoring in agricultural and environmental research as well as in industrial applications involving microorganisms. Further validation across a broader range of bacterial taxa is needed to establish the general applicability of the pipeline.

## Figures and Tables

**Table 1:** Primers and TaqMan probes used in this study.

Target (Strain, SSL or Gene)	Amplicon Length, bp	Primer	Tm, °C	Sequence 3′–5′	Reporter/Quencher
MGMM118 (SSL№10)	197	USEQ-118-F	57	GAGCGCTGTTTCTGTCGAGCG	–
		USEQ-118-R	59	GCCTGCCAACAAGACCGATAACAG	–
		USEQ-118-Z	64	ATGAGAGTCGAGATTGGTCCGGCACGCT	FAM/ BHQ-1
MGMM119 (SSL№16)	209	USEQ-119-F	57	GCGACTTGTTCCTAGTGTAATATATCAAC	–
		USEQ-119-R	58	CATGAAATATTCTCATTCTTTAATAGCCATCTC	–
		USEQ-119-Z	63	ACGAAGGCTGGGTGACAAATAATGAACACCCT	R6G/ BHQ-1
MGMM120 (SSL№18)	165	USEQ-120-F	58	TATAGCAAGTAAAACCTTAGATAGAACATCAACC	–
		USEQ-120-R	58	GGCTTTCACGAAAGATGCGTGAGC	–
		USEQ-120-Z	63	TGGCAGTGTACAGGCGTCACAGGCATACAA	TAMRA/ BHQ-2
MGMM121 (SSL№25)	190	USEQ-121-F	57	ACACAGTAATCTGATAGATTGGATCTAGG	–
		USEQ-121-R	58	CATAACGAAGGGCTGGCCGAC	–
		USEQ-121-Z	64	TAGAGGCATGTCGTCGGAATGGTTGGGAA	Cy5/ BHQ-3
*rsfS* gene [21]	-	rsfS-rt-F	60	GGCGAAGAACTGGTCGCAGTGAC	
		rsfS-rt-R	60	GGCAATGATCATGTAGTCGGTCAGGC	–
Universal primers (789–1053 16S region) [14]	-	U789-F	45	GATACCCSSGTAGTCC	–
		U1053-R	45	CTGACGRCRGCCATGC	–

**Table 2:** Mean genomic characteristics of selected strain groups (G0–G4) and numbers of SL, preSSL, and SSL revealed.

	*S. rhizophila* Group	*B. halotolerans* Group	*P. grimontii* Group	*P. viciae* Group
Size	4,444,949.8	4,119,309.2	6,766,996.2	6,661,807.2
Contigs	4.6	4	11.2	1
GC [%]	66.94	43.8	60.5	60.5
tRNAs	69	82.2	65	66.2
rRNAs	8.4	22.4	13.4	16
CDSs	4049	4056.2	6095	5746.8
pseudogenes	84.4	29.6	10.4	11.4
hypotheticals	351.6	134.4	156.4	167.2
ANIb [%]	86.49	98.9175	94.08	93.9275
Aligned [%]	72.9075	94.585	85.5625	86.205
Number of SL	111	96	105	104
Number of preSSL	20	1	12	11
Number of SSL	15	1	5	4

**Table 3:** Genes annotated in SSL ±2 kb flanking regions grouped by functional category, showing the distribution of gene products across five functional categories and their enrichment relative to genome-wide reference values (Ref %).

Functional Category	Gene Product	Count	% of Total	Ref %
DNA Replication, Repair and Modification	DNA adenine methylase (3); DNA polymerase III subunit delta’; EcoRII-like endonuclease; NUDIX hydrolase; DNA polymerase V subunit D	7	9.0%	<10% [22]
Mobile Genetic Elements and Phage-Related	SLATT-5 domain-containing protein; HNH endonuclease-like; NHH-endonuclease-like; Phage-base-V domain-containing protein; ApeA-NTD1 domain-containing protein; RHS repeat-associated protein (2); colicin E3/pyocin S6 family cytotoxin; C4-antisense RNA (3); ogr/Delta-like zinc finger containing protein; Caspase family protein; HEPN domain-containing protein; NYN domain-containing protein	15	19.2%	<5% [23]
Regulation and Signaling	Histidine kinase; DNA-binding response regulator; GGDEF domain-containing protein; Type IV pilus assembly protein PilZ; sensor domain-containing diguanylate cyclase; Competence protein J (ComJ); Bacterial nucleoid DNA-binding protein IHF-alpha; RelA-SpoT; SpoVT-AbrB domain-containing protein; Serine/threonine protein kinase; ArsR family transcriptional regulator	11	14.1%	<10% [24]
Cellular Metabolism and Biosynthesis	bifunctional aconitate hydratase; Putative metalloprotease with PDZ domain; 4-hydroxy-3-methylbut-2-enyl diphosphate reductase; signal peptidase II; isoleucine–RNA ligase; pyruvate dehydrogenase; GtrA domain-containing protein; HAD family phosphatase; HAD family hydrolase; Glycosyltransferase; Quinol monooxygenase YgiN; tRNA-Ser(gga); crotonase/enoyl-CoA hydratase family protein; rubredoxin RubB; Arsenical pump membrane protein; M24 family metallopeptidase; dTMP kinase; S49 family peptidase	18	23.1%	>35% [25]
Unknown Function or Uncharacterized	hypothetical protein (11); Various DUF domain-containing protein (10); AAA ATPase-like protein (2); sce7726 family protein; beta family protein; P63C domain protein; BIG2 domain-containing protein	27	34.6%	–

**Table 4:** Mean GC content of SSL and their ±2 kb flanking regions in each strain, compared with the corresponding whole-genome GC content.

Strain	Strain GC [%]	Fragments GC [%]	2 kb-Flanked SSL GC [%]
*S. rhizophila* MGMM118	67.1	46.4	56.7
*B. halotolerans* MGMM119	43.9	36.2	33.3
*P. grimontii* MGMM120	60.7	38	48.3
*P. viciae* MGMM121	60.7	41.9	51.3

**Table 5:** Relative transcription levels of selected SSL compared with *rsfS* in stationary-phase cultures.

Sample	Cq (SSL)	Cq (*rsfS*)	ΔCq = SSL − *rsfS*	Relative Expression, % of *rsfS*
MGMM118	24.62 ± 0.17	17.55 ± 0.51	7.07 ± 0.54	0.75 (0.51–1.10)
MGMM119	32.99 ± 0.69	25.18 ± 0.19	7.81 ± 0.72	0.45 (0.27–0.73)
MGMM120	27.96 ± 0.15	23.39 ± 0.81	4.57 ± 0.82	4.2 (2.4–7.4)
MGMM121	23.70 ± 0.23	25.11 ± 0.8	−1.41 ± 0.83	266 (149–474)

**Table 6:** Limits of detection (LOD) of the TaqMan qPCR assays with purified genomic DNA.

Sample	100 pg	10 pg	1 pg	0.1 pg	0.01 pg
MGMM118	21.23 ± 0.24	24.85 ± 0.19	28.51 ± 0.28	32.12 ± 0.33	35.20 ± 1.10
MGMM119	19.61 ± 0.06	22.99 ± 0.19	26.38 ± 0.12	29.37 ± 0.36	33.41 ± 0.81
MGMM120	26.94 ± 0.02	30.56 ± 0.12	34.24 ± 0.40	37.35 ± 0.98	-
MGMM121	24.46 ± 0.03	27.94 ± 0.06	31.44 ± 0.21	34.95 ± 0.07	37.10 ± 0.22

**Table 7:** Method detection limits of the qPCR assay for target strains in soil.

Strain	MDL (CFU/g)	Genome Copies per CFU (Mean ± SD)
MGMM118	1.4 × 10^4^	0.66 ± 0.03
MGMM119	6.5 × 10^3^	0.48 ± 0.10
MGMM120	1.5 × 10^4^	2.12 ± 0.43
MGMM121	1.3 × 10^4^	0.52 ± 0.10

Note: Genome copies per CFU were calculated for each dilution as the ratio of qPCR-measured genome equivalents to the number of inoculated CFU. Detailed data per dilution are provided in Table S7. MDL is the lowest CFU/g at which target DNA was detected in all three biological replicates.

**Table 8:** Detection of the target strains in winter wheat rhizosphere seven days after consortium application.

Sample	Cq (Mean ± SD)	DNA per Reaction (pg, Mean ± SD)	DNA Copies per Reaction Mean ± SD)	Calculated CFU/g
MGMM118	23.07 ± 0.12	31.19 ± 2.29	6780 ± 498	3.9 × 10^6^
MGMM119	25.71 ± 0.17	1.44 ± 0.17	321 ± 38	2.5 × 10^5^
MGMM120	26.86 ± 0.29	113.56 ± 22.23	16,223 ± 3176	2.9 × 10^6^
MGMM121	24.82 ± 0.09	78.30 ± 4.68	11,029 ± 659	8.0 × 10^6^

Note: CFU/g was calculated using the genome-copies-per-CFU conversion coefficient (Table 7) and normalized to 1 g of soil.

## Data Availability

The original contributions presented in this study are included in the article. Further inquiries can be directed to the corresponding author.

## Supplementary Materials

The following supporting information can be downloaded at: https://www.mdpi.com/article/10.3390/microorganisms14071587/s1, Table S1: General genome features of the four target strains used for SSL discovery; Table S2: Sequences and characteristics of all identified SSL; Table S3: Target strains and their closest non-target genomes used for KEC filtering, as determined by TCS and BLASTn analyses; Table S4: Genetic context in 2 kb SSL flanking regions and nearest annotated genes; Table S5: PCR amplicons around SSL targets and their characteristics; Figure S1: RT-qPCR of SSL transcription and assessment of genomic DNA contamination; Table S6: Calibration curve parameters of the strain-specific TaqMan qPCR assays; Figure S2: Analytical sensitivity of SSL-based qPCR assays on purified genomic DNA; Table S7: Spike-in experimental data for determination of strain-specific DNA extraction efficiency and genome copies per CFU in soil; Table S8: Soil samples description, used for specificity test.

## Abbreviations

The following abbreviations are used in this manuscript:

SL	Specific loci
preSSL	Presumed strain specific loci
SSL	Strain specific loci

## References

[1] Briczinski E.P., Loquasto J.R., Barrangou R., Dudley E.G., Roberts A.M., Roberts R.F. (2009). Strain-Specific Genotyping of *Bifidobacterium animalis* subsp. *lactis* by Using Single-Nucleotide Polymorphisms, Insertions, and Deletions. Appl. Environ. Microbiol..

[2] Andronov E.E., Aksenova T.S., Onishchuk O.P., Kurchak O.N., Safronova V.I., Pinaev A.G., Evsyukov I.V., Provorov N.A. (2025). Strain-Specific Markers of Rhizobia According to Whole Genome Sequencing Data. Microbiology.

[3] Jo B.-H., Lee C.S., Song H.-R., Lee H.-G., Oh H.-M. (2014). Development of Novel Microsatellite Markers for Strain-Specific Identification of Chlorella Vulgaris. J. Microbiol. Biotechnol..

[4] Hernández I., Sant C., Martínez R., Fernández C. (2020). Design of Bacterial Strain-Specific qPCR Assays Using NGS Data and Publicly Available Resources and Its Application to Track Biocontrol Strains. Front. Microbiol..

[5] Wang X., Tian X., Li W., Yang Y., Zhang S., Wang H., Geng W., Zhai J. (2025). An SNP-Based Diagnostic Method for Brucella S2 Vaccine Strain Infections. Front. Vet. Sci..

[6] Louws F., Rademaker J., De Bruijn F. (1999). THE THREE DS OF PCR-BASED GENOMIC ANALYSIS OF PHYTOBACTERIA: Diversity, Detection, and Disease Diagnosis. Annu. Rev. Phytopathol..

[7] He X., Zeng M., Bai W., Tang Z., Ding J., Chen Z. (2025). Rapid On-Site Detection of Pseudomonas Aeruginosa via ecfX-Targeted Loop-Mediated Isothermal Amplification. Biosensors.

[8] Chambers G.K., MacAvoy E.S. (2000). Microsatellites: Consensus and Controversy. Comp. Biochem. Physiol. B Biochem. Mol. Biol..

[9] Ghezzi H., Fan Y.M., Ng K.M., Burckhardt J.C., Pepin D.M., Lin X., Ziels R.M., Tropini C. (2024). PUPpy: A Primer Design Pipeline for Substrain-Level Microbial Detection and Absolute Quantification. mSphere.

[10] Kodama Y., Shumway M., Leinonen R., on behalf of the International Nucleotide Sequence Database Collaboration (2012). The Sequence Read Archive: Explosive Growth of Sequencing Data. Nucleic Acids Res..

[11] Teeling H., Meyerdierks A., Bauer M., Amann R., Glöckner F.O. (2004). Application of Tetranucleotide Frequencies for the Assignment of Genomic Fragments. Environ. Microbiol..

[12] Konstantinidis K.T., Tiedje J.M. (2005). Genomic Insights That Advance the Species Definition for Prokaryotes. Proc. Natl. Acad. Sci. USA.

[13] Beran P., Stehlíková D., Cohen S.P., Čurn V. (2021). KEC: Unique Sequence Search by K-Mer Exclusion. Bioinformatics.

[14] Brunk C.F., Li J., Avaniss-Aghajani E. (2002). Analysis of Specific Bacteria from Environmental Samples Using a Quantitative Polymerase Chain Reaction. Curr. Issues Mol. Biol..

[15] Boodman C., Gupta N., Cimen C., Van Griensven J., Cheng M.P., Yansouni C.P., Bottieau E. (2026). Etiologies of Community-Acquired Febrile Illness Identified by TaqMan Array Card qPCR on Blood Samples: A Systematic Review and Meta-Analysis. J. Clin. Microbiol..

[16] King E.O., Ward M.K., Raney D.E. (1954). Two Simple Media for the Demonstration of Pyocyanin and Fluorescin. J. Lab. Clin. Med..

[17] Camacho C., Coulouris G., Avagyan V., Ma N., Papadopoulos J., Bealer K., Madden T.L. (2009). BLAST+: Architecture and Applications. BMC Bioinform..

[18] Mistry J., Chuguransky S., Williams L., Qureshi M., Salazar G.A., Sonnhammer E.L.L., Tosatto S.C.E., Paladin L., Raj S., Richardson L.J. (2021). Pfam: The Protein Families Database in 2021. Nucleic Acids Res..

[19] Wishart D.S., Han S., Saha S., Oler E., Peters H., Grant J.R., Stothard P., Gautam V. (2023). PHASTEST: Faster than PHASTER, Better than PHAST. Nucleic Acids Res..

[20] Jiang H., Lei R., Ding S.-W., Zhu S. (2014). Skewer: A Fast and Accurate Adapter Trimmer for next-Generation Sequencing Paired-End Reads. BMC Bioinform..

[21] Miftakhov A.K., Diabankana R.G.C., Frolov M., Yusupov M.M., Validov S.Z., Afordoanyi D.M. (2022). Persistence as a Constituent of a Biocontrol Mechanism (Competition for Nutrients and Niches) in Pseudomonas Putida PCL1760. Microorganisms.

[22] Vanni C., Schechter M.S., Acinas S.G., Barberán A., Buttigieg P.L., Casamayor E.O., Delmont T.O., Duarte C.M., Eren A.M., Finn R.D. (2022). Unifying the Known and Unknown Microbial Coding Sequence Space. eLife.

[23] Newton I.L.G., Bordenstein S.R. (2011). Correlations Between Bacterial Ecology and Mobile DNA. Curr. Microbiol..

[24] De Lazzari E., Grilli J., Maslov S., Cosentino Lagomarsino M. (2017). Family-Specific Scaling Laws in Bacterial Genomes. Nucleic Acids Res..

[25] Zeng Q., Xie J., Li Y., Gao T., Xu C., Wang Q. (2018). Comparative Genomic and Functional Analyses of Four Sequenced Bacillus Cereus Genomes Reveal Conservation of Genes Relevant to Plant-Growth-Promoting Traits. Sci. Rep..

[26] Sharma G.K., Sharma R., Joshi K., Qureshi S., Mathur S., Sinha S., Chatterjee S., Nunia V. (2024). Advancing Microbial Diagnostics: A Universal Phylogeny Guided Computational Algorithm to Find Unique Sequences for Precise Microorganism Detection. Brief. Bioinform..

[27] Haubold B., Klötzl F., Hellberg L., Thompson D., Cavalar M. (2021). Fur: Find Unique Genomic Regions for Diagnostic PCR. Bioinformatics.

[28] Tsifintaris M., Koutra P., Tsiartas P., Repanas P., Touliopoulos S., Nelios G., Anastasiadou A., Tamouridou G., Nikolaou A., Tsochantaridis I. (2026). Erimin: A Pipeline to Identify Bacterial Strain Specific Primers. DNA.

[29] Vernikos G., Medini D., Riley D.R., Tettelin H. (2015). Ten Years of Pan-Genome Analyses. Curr. Opin. Microbiol..

[30] SantaLucia J., Hicks D. (2004). The Thermodynamics of DNA Structural Motifs. Annu. Rev. Biophys. Biomol. Struct..

[31] Sakoparnig T., Field C., Van Nimwegen E. (2021). Whole Genome Phylogenies Reflect the Distributions of Recombination Rates for Many Bacterial Species. eLife.

[32] Hershberg R., Petrov D.A. (2010). Evidence That Mutation Is Universally Biased towards AT in Bacteria. PLoS Genet..

[33] Koonin E.V., Makarova K.S., Wolf Y.I. (2017). Evolutionary Genomics of Defense Systems in Archaea and Bacteria. Annu. Rev. Microbiol..

[34] Kibby E.M., Whiteley A.T. (2020). The Linguistics of Bacterial Conflict Systems Reveal Ancient Origins of Eukaryotic Innate Immunity. J. Bacteriol..

[35] Navarre W.W., Porwollik S., Wang Y., McClelland M., Rosen H., Libby S.J., Fang F.C. (2006). Selective Silencing of Foreign DNA with Low GC Content by the H-NS Protein in *Salmonella*. Science.

[36] Duan B., Ding P., Navarre W.W., Liu J., Xia B. (2021). Xenogeneic Silencing and Bacterial Genome Evolution: Mechanisms for DNA Recognition Imply Multifaceted Roles of Xenogeneic Silencers. Mol. Biol. Evol..

[37] Plaire D., Puaud S., Marsolier-Kergoat M.-C., Elalouf J.-M. (2017). Comparative Analysis of the Sensitivity of Metagenomic Sequencing and PCR to Detect a Biowarfare Simulant (Bacillus Atrophaeus) in Soil Samples. PLoS ONE.

[38] Pecoraro V., Zerulla K., Lange C., Soppa J. (2011). Quantification of Ploidy in Proteobacteria Revealed the Existence of Monoploid, (Mero-)Oligoploid and Polyploid Species. PLoS ONE.

[39] Böttinger B., Semmler F., Zerulla K., Ludt K., Soppa J. (2018). Regulated Ploidy of Bacillus Subtilis and Three New Isolates of Bacillus and Paenibacillus. FEMS Microbiol. Lett..

[40] Wielgoss S., Barrick J.E., Tenaillon O., Cruveiller S., Chane-Woon-Ming B., Médigue C., Lenski R.E., Schneider D. (2011). Mutation Rate Inferred from Synonymous Substitutions in a Long-Term Evolution Experiment with Escherichia Coli. G3.

[41] Drake J.W., Charlesworth B., Charlesworth D., Crow J.F. (1998). Rates of Spontaneous Mutation. Genetics.

