# Supplementary material for: Strain-Specific Loci in Bacterial Genomes: Whole-Genome Discovery, Genomic Context, and Application for Multi-Strain qPCR Monitoring
Source: Microorganisms. 2026 Jul 21;14(7):1587. doi: 10.3390/microorganisms14071587 (PMC13414232; doi:10.3390/microorganisms14071587)
Supplement: Supplementary file 1 [file microorganisms-14-01587-s001.zip › microorganisms-4424890-supplementary.pdf]

Supplementary materials

Table S1. General genome features of the four target strains used for SSL discovery.

**Genome**	***S. rhizophila*** **MGMM118**	***B. halotolerans*** **MGMM119**	***P. grimontii*** **MGMM120**	***P. viciae*** **MGMM121**
**Size, bp**	4171692	4072052	6373430	6466577
**Contigs**	1	1	1	1
**Completeness [%]**	98.04	98.82	100	99.95
**Contamination [%]**	1.16	0.29	0.83	0.19
**GC [%]**	67.1	43.9	60.7	60.7
**tRNAs**	69	86	68	67
**rRNAs**	10	30	19	16
**ncRNAs**	13	24	57	89
**CDSs**	3666	3931	5701	5571
**pseudogenes**	4	21	13	8
**hypotheticals**	166	96	123	159

Table S2. Sequences and characteristics of all identified SSL.

Strain (GC%)	SSL №	% GC	Length (bp)	Coding (C) or not (N)	BLAST match E-value	Sequence
**MGMM 118 (67,1)**	1	49	125	C	>3	ccgtcttggagaataacgaaaccacatcaaagaagttgggcgttgcgagttcgtagtagtacgacccattgcttgcagtctttcgtgagcactctgatgcgtgtatgccactcctatagtgacgt
	2-4	40	120	N	0.53	agcagaagttactaaaaaagaacctatgctcgataaccacatcaatggattaactaaccgcaggagatcgacagacgctcaatttgcgacaaatagttcgaaattcacgacaatcgaaca
		38	235	C/N	0.15	acagccaattcaactagacccaccccaatcacccatcaaaaattgtgatgcaactttagatcaaaacaacattaataaaagtcacccgagcctcgaaggcagagtaatttaaaaatcttaaaataaccaacaatcgaaacataaaatttcgcgcattagggcagcagataacctgcgaatacatcaatcccatcaactattcactaaacttctacgaaacagacttccgagaagc
		44	175	C/N	>3	ctatcgccactggaaccaagacgccaagaaaagccagaataccgaaagtcacaaccatgaggcggttaaatgcctgatcgtaaaattggtttacagaagacagtagctgaattgaatccatttcaccctcgcaaacgtcctattcttttaaccacttccaaagccataacgacga
	5-8	47	129	C	1.9	gtggttgtgattaacctatggcgcaactcgtcgggtcaacacctaaactgaattaacttcgcactttttgactattcctatatttcaattcttattttctgcaaagtacttcgcactatgtgggatttt
		48	134	C	2	ctcatccaatagcactgtcttcacatacctgtccatatcgtccttctgtgccattaagtaggacggaagacgggatgtcaaaggtgattccaaaacgatcttgaaggacgttagctcacgcagctcttgatggg
		46	140	C	0.7	cgtcgaggcttctgcatctttaagcgcggcctctatcgcttcaaagcttgcaagaccatcgataagctgaacacccatagtaatgtcgatatttatcgaatcgcccgctatggattcaaaaaccaagcttttaatccgca
		51	150	C	2.4	tcgatcccactttttgagatgagcgtagtacgtccccgctgtatttgcgcacaggtccgcactattaaccagcgacgcaaccaaatgacactcttctgccttggaaagaagctcctcttccttccaagacttgagggcatgccatatgcc
	9	47	129	C	>3	aattcgaaatgaactccacttgcccaaatggaattgaacaaaagccgaggcttgtattgggagagagccaaggttgaatcaaacaccccgctagcaattccgatctcacgaaagacagtagcctcatcg
	10-15	50	142	N	>3	atgatcagattctcgttagcgtgccggaccaatctcgactctcatcctcttcttccgtggaatcatttaggagcgaatttgagggagccctctcaggcttgtacgacgcaagtcgacatctgacgttcgtcctagatgtaca
		49	159	C	2.6	ttccctttggtgactcgtacacgcttatcggctttgagttccctacagatgaggtgaagggaaagcttacgattaatgcgattgttggcccaaacggaactggaaagacgcgaacgctcgtttctcttgcaaagtgcacagccgattctagccaagagt
		49	211	C	>3	gttcctgaatcacctacgcaagctcgctatcgaatctagagcagaaggtgttattcagacattgtggggcaacctaccgttgatacttcgtgaactcagaactcctccagattcagactcggtatcagacgagatcagacgactgtgcgtcacctacaagcacaattgcggagaagcctcagttcctgcattgttcttcaaggctttgagc
		51	130	C	>3	ctggagtcatacccttttagcgcggagtagacaacttgcgagctctcgagatcggagaaatcttctccatcaagcgagagttgcatcggaatgtcatcatcccaagcaacggtgggaacgtcaatgttcca
		50	137	C	>3	ggtgggtaagcaacggtgcgtagagaaggttccgaatggtccccccactcgacagactcccacttctaattgaggtaatggtgctgaacatatcctccgatacgccaatgaccactttatcgttgtaaacatccaca
		50	129	C/N	0.61	cgtctcaaccccggagtatcccaggccctcaaccttacgggcttgattttctaagaatttgatcataaacatccctattcattgtttcggatagcctgctaggtggccccttgcgacctgaggtacgaa

**MGMM 119 (43,9)**	16	36	174	N	0.12	tataacgaaccaatggtttaccgaatttggaaatagttcatataggagctaataatgtgacgccatcagtgatgattatgaacgctacagcgacttgttcctagtgtaatatatcaacatataaggatgatagacaaagggcatataatggaggttttactcccagagtgaaaa
**MGMM 120 (60,7)**	17	49	163	C/N	0.88	tttatgatttaataatttgcaatccccgattatagagggcaacacttaaatatctattagcagcccgactcacttacacgacaattatatggaaataaaattatgacagatacgccaattagccaacctagcgttgataaattcataacacgtatctctggaa
	18	41	187	C/N	0.34	ggatgctgcagctagcattaaacctatagcaagtaaaaccttagatagaacatcaacctaactggcagtgtacaggcgtcacaggcatacaacactccttacccccaatgacaacatgtacattaaataagaaacttcagagcaccactgaatagtgaatttatagctcacgcatctttcgtgaaag
	19	35	185	C/N	0.34	tgaagctattttacctgcacttatctgagcaacctattaaagaccatgaactttaaactcagaaagttagcgactaagcaatgaccgctgtagatctaattaaaaccttgtcaaagaatctatttactacaaacaacctagtataaaaggggcggatttatttagatcaacggccataagtaaaa
	20	38	175	C/N	0.31	atctcgatagtgagaatatcgaataacaggaatatttacttcagtgatagcaactgaagtatacgagatctcttagagttggtctttaaagtttctcttgttttagtgtgcgtggaagctagtcaataggatatgcgttagtggttgtgtttagactgaagtcaactactccgct
	21*	44	121	C/N	0.54	ggtttgtctagtggggcgaatgagatacggtctacttggtcggatacgtgtagaaaatgatataaatgctaagtatttttagccggtcggaacgctgatgcgagtctattgcaagatctgt
**MGMM 121 (60,7)**	22	36	132	C	0.2	tgccaatatgcatgaggtattaaaggcgactaaaaaatatcacgaaataaaagttctcgacgttatgtcggcaggagaagttaaaaaaactctgcgggagtttttttctagatttactagcacttcaagtat
	23	34	144	C/N	0.23	aacataaagcgagtttctgttataagcttctgttagttgtaattaaaattataaggaagggagtttaaatgggaattgataaaaaaattcactttcccttttggtagaagctttatgcttcgagccgaactaggtcaaggatcgt
	24	46	140	C	0.23	gtaatgcggggggtagatttgagattgtagcggagggaagcaatcactacgggttgctccattctccttccattcgcatagcacgatataaccctccaaccaatttactcagggataaaacatatgagtttagtctgcgt
	25	57	135	N	0.65	tgaccaccggacgtcgatctgctaaagaccgttcccaaccattccgacgacatgcctctatcaagaggaatgcttccaacgagcgctcaacgctcgcgcagtcagtagaactgtcatcggtcggccagcccttcg

Underlined sequences indicate regions for which primers and the probe were designed for TaqMan qPCR detection.

* located within a prophage region predicted by PHASTEST.

Table S3. Target strains and their closest non-target genomes used for KEC filtering, as determined by TCS and BLASTn analyses.

Target genome	Non-target genomes		ANIb [%]	Aligned [%]	Aligned/total [bp]
	Name	RefSeq Number			
***S. rhizophila* MGMM118**	*S. sp.* BIO128-B (SL BLAST**)	GCF_030128875.1	91,32	79,52	3317305/4171692
	S. rhizophila THA 2.2 (SL BLAST)	GCF_000661955.1	85,48	73,8	3078534/4171692
	S. nematodicola CPCC 101271 (TCS*)	GCF_009467805.1	84,96	70,53	2942240/4171692
	S. rhizophila QL-P4 (TCS)	GCF_001704155.1	84,2	67,78	2827410/4171692
***B. halotolerans* MGMM119**	*B. halotolerans* XE48 (SL BLAST)	GCF_025809355.1	99,05	96,34	3923108/4072052
	B. halotolerans ATCC25096 (TCS)	GCF_001517105.1	98,89	93,82	3820317/4072052
	B. halotolerans FJAT-2398 (TCS)	GCF_001637525.1	98,89	93,95	3825823/4072052
	B. halotolerans XYK2-4 (SL BLAST)	GCF_041222785.1	98,84	94,23	3837008/4072052
***P.grimontii* MGMM120**	*P. marginalis* MGMM3 (SL BLAST)	GCF_029916865.1	94,45	86,88	5537293/6373430
	P. marginalis M13 (TCS)	GCF_045037965.1	94,13	86,49	5512390/6373430
	P. grimontii DSM 17515 (TCS)	GCF_900101085.1	93,8	84	5353959/6373430

	P. grimontii BIGb0428 (SL BLAST)	GCF_024807865.1	93,94	84,88	5409668/6373430
***P. viciae* MGMM121**	*P. brassicacearum* DF41 (TCS)	GCF_000585995.1	98,85	94,31	6098710/6466577
	P. sp. B21-054 (SL BLAST)	GCF_026016345.1	94,4	85,24	5512240/6466577
	P. sp. G2-4 (SL BLAST)	GCF_030064125.1	92,84	84,83	5485871/6466577
	P. viciae 11K1 (TCS)	GCF_004786035.1	89,62	80,44	5201953/6466577

* strains identified by TCS.

** strains identified from BLASTn analysis of Specific Loci (SL).

Table S4. Genetic context in 2 kb SSL flanking regions and nearest annotated genes.

Strain	SSL ID	GC content (%)	Length of SSL flanking region (bp)	Total length of coding sequences (bp)	Nearest annotated ORF	Distance to SSL (bp)
**MGMM118**	SSL1	60	4125	2798	bifunctional aconitate hydratase	1380
					RHS repeat-associated protein	0
	SSL2-4 (cluster)	61	5636	4329	Putative metalloprotease with PDZ domain	165
					hypothetical protein	0
					isoleucine--tRNA ligase	1610
					4-hydroxy-3-methylbut-2-enyl diphosphate reductase	101
					signal peptidase II	1105
	SSL5-8 (cluster)	55	10216	9479	Histidine kinase	1842
					DNA-binding response regulator	1145
					SLATT-5 domain-containing protein	334
					site-specific DNA-methyltransferase (adenine-specific)	0
					hypothetical protein	449
					GGDEF domain-containing protein	0
					HAD family phosphatase	491
					site-specific DNA-methyltransferase (adenine-specific)	0
					DNA polymerase III subunit delta'	1007
					Type IV pilus assembly protein PilZ	647
					dTMP kinase	1958
	SSL9	57	4129	3974	hypothetical protein	0
					Glycosyltransferase	272
					GtrA domain-containing protein	1705
	SSL10-15 (cluster)	55	10353	8580	pyruvate dehydrogenase (acetyl-transferring)	504
					AAA ATPase-like	0
					NHH endonuclease-like	0
					DUF6339 domain-containing protein	0
					hypothetical protein	0
					hypothetical protein	0
					EcoRII-like endonuclease	139
**MGMM119**	SSL16	33	4174	2557	sce7726 family protein	1418
					beta family protein	492
					P63C domain protein	477
					Quinol monooxygenase YgiN	1822

**MGMM120**	SSL17	51	4187	3059	Phage-base-V domain-containing protein	1244
					DUF3596 domain-containing protein	0
					hypothetical protein	79
					tRNA-Ser(gga)	1540
					crotonase/enoyl-CoA hydratase family protein	1966
	SSL18	44	4163	2667	sensor domain-containing diguanylate cyclase	1394
					AAA family ATPase-like	0
					HNH endonuclease	1198
	SSL19	52	4185	3309	RHS repeat-associated protein	1713
					DUF4304 domain-containing protein	1094
					DUF4304 domain-containing protein	0
					hypothetical protein	51
					Competence protein J (ComJ)	124
					Bacterial nucleoid DNA-binding protein IHF-alpha	623
					rubredoxin RubB	1090
	SSL20	44	4175	3721	RelA-SpoT domain-containing protein	0
					DUF3800 domain-containing protein	0
					DUF805 domain-containing protein	1324
					DUF4747 domain-containing protein	1919
	SSL21	51	4121	3388	DUF91 domain-containing protein	1562
					ApeA-NTD1 domain-containing protein	50
					ogr/Delta-like zinc finger family protein	0
					hypothetical protein	209
					SpoVT-AbrB domain-containing protein	778
					NUDIX hydrolase	1220
					hypothetical protein	1468
**MGMM121**	SSL22	49	4132	3892	C4-antisense RNA	87
					colicin E3/pyocin S6 family cytotoxin	1073
					DUF6124 family protein	224
					Caspase domain-containing protein	0
					HEPN domain-containing protein	1454
	SSL23	46	4144	2827	hypothetical protein	1469
					hypothetical protein	733
					NYN domain-containing protein	457
					Serine/threonine protein kinase	0
					HAD family hydrolase	1144

	SSL24	58	4140	3596	ArsR family transcriptional regulator	1208
					C4-a1b1-antisense RNA	443
					C4-antisense RNA	541
					BIG2 domain-containing protein	0
					DUF6124 domain-containing protein	683
					Arsenical pump membrane protein	1596
	SSL25	52	4135	3035	M24 family metallopeptidase	777
					S49 family peptidase	470
					site-specific DNA-methyltransferase (adenine-specific)	1359
					DNA polymerase V subunit D	126

The table shows, for each strain-specific locus (SSL), the GC content and length of the window (SSL plus ±2 kbp), the total length of coding sequences within this window, and the nearest annotated ORFs with their distances to the SSL (in base pairs). This information forms the basis for functional classification of genes in the SSL genomic environment.

Table S5. PCR amplicons around SSL targets and their characteristics.

Strain	Sequence	Length, bp	%GC
MGMM118	**gcctgccaacaagaccgataacag**catgaatgatcagattctcgtt**agcgtgccggaccaatctcgactctcat**cctcttcttccgtggaatcatttaggagcgaatttgagggagccctctcaggcttgtacgacgcaagtcgacatctgacgttcgtcctagatgtacagggcc**cgctcgacagaaacagcgctc**	197	53
MGMM119	**gcgacttgttcctagtgtaatatatcaac**atataaggatgatagacaaagggcatataatggaggttttactcccagagtgaaaaagtagagagagaaagattagacaatgaaggatatattcaatagg**acgaaggctgggtgacaaataatgaacaccct**cttataattaaagtg**gagatggctattaaagaatgagaatatttcatg**	209	35
MGMM120	**tatagcaagtaaaaccttagatagaacatcaacc**taac**tggcagtgtacaggcgtcacaggcatacaa**cactccttacccccaatgacaacatgtacattaaataagaaacttcagagcaccactgaatagtgaatttata**gctcacgcatctttcgtgaaagcc**	165	41
MGMM121	**acacagtaatctgatagattggatctagg**tcaatttttgtgggaagaatctgaccaccggacgtcgatctgctaaagaccg**ttcccaaccattccgacgacatgcctcta**tcaagaggaatgcttccaacgagcgctcaacgctcgcgcagtcagtagaactgtcatcgg**gtcggccagcccttcgttatg**	190	51

The table summarizes the amplicons generated by the SSL-targeted qPCR assays, including sequence length, and GC content. Amplicon regions may be shifted relative to the original SSL due to primer design constraints. Bold and underlined nucleotides indicate the binding sites of primers and TaqMan probes.

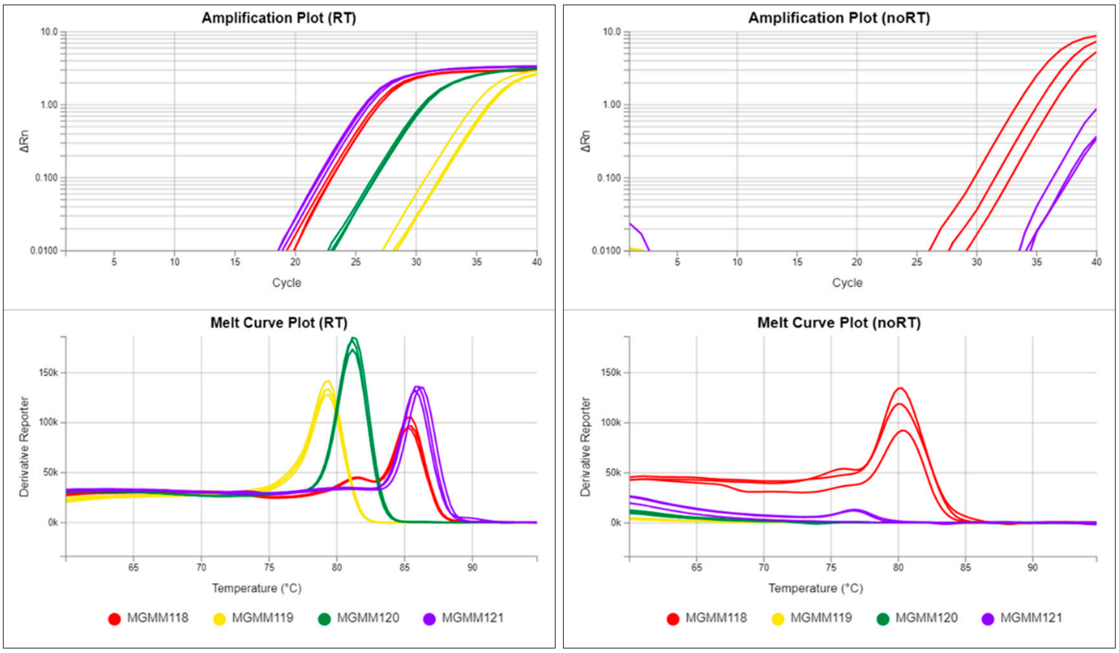

Figure S1. RT-qPCR of SSL transcription and assessment of genomic DNA contamination.

Left panel: amplification curves for SSL and *rsfS* in cDNA samples obtained after reverse transcription (RT). **Right panel**: amplification curves for no-RT controls (RNA samples without reverse transcriptase). Late, heterogeneous amplification in no-RT samples, combined with melt-curve analysis, indicates non-specific amplification or minor contamination rather than true genomic DNA carryover.

Table S6. Calibration curve parameters of the strain-specific TaqMan qPCR assays.

Strain	Slope	R^2	Efficiency (%)
MGMM118	–3.632	1	88.5
MGMM119	–3.269	0.9991	102.3
MGMM120	–3.493	0.9986	93.4
MGMM121	–3.499	1	93.1

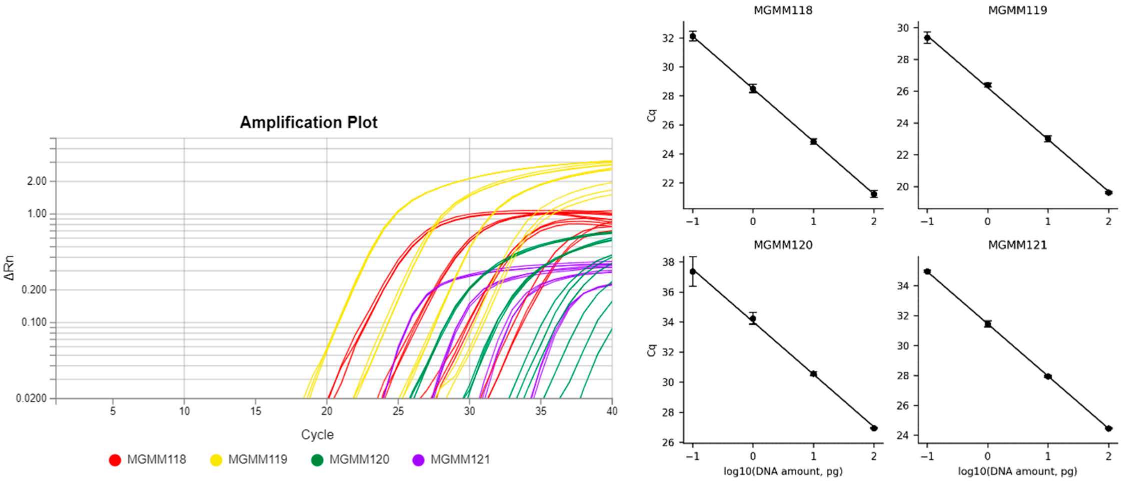

Figure S2. Analytical sensitivity of SSL-based qPCR assays on purified genomic DNA.

Left: amplification plots for ten-fold serial dilutions of genomic DNA (100–0.01 pg per reaction) for the four TaqMan assays. **Right**: standard curves constructed from the lowest DNA amounts tested, showing linear relationships between log(DNA amount) and Cq over the quantitative range used to define LOQ.

Table S7. Spike-in experimental data for determination of strain-specific DNA extraction efficiency and genome copies per CFU in soil.

Strain	OD_{600}	Inoculated CFU	Extracted DNA (pg, mean ± SD)	Detected genome copies (mean ± SD)	Genome copies per CFU
S. rhizophila MGMM118	0.01	560 000	1602.19 ± 192.97	350 400 ± 42 200	0.63 ± 0.08
	0.001	56 000	167.74 ± 18.08	36 800 ± 3 900	0.66 ± 0.07
	0.0001	5 600	17.56 ± 10.80	3 900 ± 2 400	0.70 ± 0.42
B. halotolerans MGMM119	0.01	65 000	147.64 ± 13.52	33 200 ± 3 100	0.51 ± 0.05
	0.001	6 500	16.16 ± 0.68	3 590 ± 220	0.55± 0.03
	0.0001	650	1.09 ± 0.67	250 ± 160	0.38 ± 0.22
P. grimontii MGMM120	0.01	600 000	9892.28 ± 972.12	1 416 200 ± 139 200	2.36 ± 0.23
	0.001	60 000	997.77 ± 206.30	142 900 ± 29 500	2.38 ± 0.49
	0.0001	6 000	68.11 ± 30.39	9 800 ± 4 400	1.63 ± 0.73
P. viciae MGMM121	0.01	520 000	1594.27 ± 220.31	224 900 ± 31 000	0.43 ± 0.06
	0.001	52 000	233.39 ± 10.75	32 900 ± 1 600	0.63 ± 0.03
	0.0001	5 200	18.90 ± 6.72	2 670 ± 950	0.51 ± 0.18

Genome copies were calculated as extracted DNA (pg) divided by the theoretical mass of a single genome copy (genome size in bp × 1.096×10^{-3} fg/bp). Genome copies per CFU for each dilution were obtained as genome copies divided by inoculated CFU.

Table S8. Soil samples description, used for specificity test.

No.	Location	Coordinates	Description
1	Russia, Republic of Tatarstan, Laishevsky District	55°37′17.8″N 49°19′34.2″E	Three sampling points from the field where the target strains were originally isolated
2	Russia, Republic of Tatarstan, Laishevsky District		
3	Russia, Republic of Tatarstan, Laishevsky District		
4	Russia, Republic of Tatarstan, Laishevsky District	55°37′23.7″N 49°19′40.8″E	Sampling site located ~500 m from the original isolation site
5	Russia, Republic of Tatarstan, Laishevsky District	55°37′41.9″N 49°20′27.1″E	Sampling site located ~1000 m from the original isolation site
6	Russia, Republic of Tatarstan, Kazan	55°45′32.0″N 49°08′45.5″E	Roadside forest belt within city limits
7	Russia, Republic of Tatarstan, Stary Yumoraly village	55°07′42.9″N 48°35′17.8″E	Ploughed agricultural field
8	Russia, Sverdlovsk Oblast	59°58′28.7″N 59°47′19.8″E	The Ural Mountains
9	6 Tràng Tiền, Hoàn Kiếm, Hanoi, Vietnam	21°01′30.6″N 105°51′34.5″E	Hanoi public park
10	—	—	Non-sterile commercial potting soil (Nord Pulp LLC, Russian Federation)
